# Utilisation of mobile phone interventions to improve the delivery of maternal health services in sub-Saharan Africa: A scoping review protocol

**DOI:** 10.1371/journal.pone.0295437

**Published:** 2024-03-06

**Authors:** Asahngwa Constantine, Arone Wondwossen Fantaye, Amos Buh, Pamela Obegu, Karine Fournier, Mwenya Kasonde, Choolwe Jacobs, Phiri Clementina, Ronald Gobina, Odette Kibu, Denis Foretia, Miriam Nkangu, Sanni Yaya

**Affiliations:** 1 Nkafu Policy Institute, Denis and Lenora Foretia Foundation Cameroon, Yaounde, Cameroon; 2 Faculty of Medicine, University of Ottawa, Ottawa, Ontario, Canada; 3 Interdisciplinary School of Health Sciences, University of Ottawa, Ottawa, Ontario, Canada; 4 Bruyere Research Institute, Ottawa, Ontario, Canada; 5 Health Sciences Library, University of Ottawa, Ottawa, Ontario, Canada; 6 Liverpool School of Tropical Medicine, Liverpool, United Kingdom; 7 School of Public Health, University of Zambia, Lusaka, Zambia; 8 Ministry of Health, Lusaka, Zambia; 9 Center for Multicultural and Global Health, University of Tennessee Health Science Center, Memphis, TN, United States of America; 10 School of Epidemiology and Public Health, University of Ottawa, Ottawa, Ontario, Canada; 11 School of International Development and Global Studies, University of Ottawa, Ottawa, Ontario, Canada; 12 The George Institute for Global Health, Imperial College London, London, United Kingdom; Independent Consultant, UNITED STATES

## Abstract

**Introduction:**

There has been significant progress in maternal health outcomes in the sub-Saharan African region since the early 1990s, in part due to digital and mobile health interventions. However, critical gaps and disparities remain. Mobile phones in particular have potential to reach underserved, hard-to-reach populations with underdeveloped infrastructure. In spite of the opportunities for mobile phones to address maternal mortality in the region, there is no extensive mapping of the available literature on mobile phone interventions that aim to improve access of maternal care in sub-Saharan Africa. The proposed scoping review aims to map literature on the nature and extent of mobile phones interventions designed to improve maternal care health services in the region.

**Methods:**

Conduct of this scoping review will be guided by the Joanna Briggs Institute approach. Literature searches will be conducted in multiple electronic databases, including MEDLINE, Embase, CINAHL, APA PsycInfo, Cochrane Central Register of Controlled Trials, Global Health, African Index Medicus, Web of Science, and Applied Social Sciences Index & Abstracts. Grey literature will also be identified. Keyword searches will be used to identify articles. Two reviewers will independently screen eligible titles, abstracts and full articles with a third reviewer to help resolve any disputes. We will extract data on general study characteristics, population characteristics, concept, context, intervention details, study results, gaps and recommendations.

**Discussion:**

Understanding use of mobile phones among underserved, hard-to-reach populations with underdeveloped infrastructure to address maternal mortality in developing countries is very critical to informing health systems on potential effective strategies. This review will complement the evidence base on utilisation of mobile phone interventions to improve the delivery of maternal health services in sub-Saharan Africa.

## Introduction

Maternal health refers to women’s health and well-being during the periods of pregnancy, childbirth and puerperium [1]. Since the adoption of the Declaration of Alma-Ata in 1978, maternal health has become a ubiquitous priority among countless national and international stakeholders, including governments, civil society, multilateral organisations, and the private sector [2, 3]. This priority was emphasized by global commitments and efforts to meet the primary aims of the 1987 Safe Motherhood Initiative [4], and the targets of the fifth Millennium Development Goal (MDG) [5]. Maternal health remained a key priority in the 2030 Agenda for Sustainable Development, through the Sustainable Development Goals (SDGs), as it received major coverage in the third and fifth SDG, to ensure healthy lives and promote well-being for all at all ages and to achieve gender equality and empower all women and girls, respectively [6, 7]. In the last 20 years, several global initiatives have been developed to help fast-track progress towards maternal health targets set in the MDGs and SDGs [2, 8–10]. The development goals and global initiatives galvanized major advocacy, political commitments, and greater financial investments into the annual development assistance for maternal health [9, 11, 12]. This is particularly important as the coronavirus pandemic has hindered progress towards the attainment of SDGs further [13]. The Goals and initiatives have also encouraged the development and implementation of local and national community-based and health facility-based interventions across the continuum of maternal healthcare [2, 12, 14]. These interventions range from training and linking traditional birth attendants to a health system [15], to establishing maternity waiting homes [16] and mobile maternal health services [17].

Despite the growing number of global initiatives and meso and micro-level interventions aimed at reducing adverse maternal health outcomes, there remain critical gaps and disparities in access to maternal health services in low and middle income countries (LMICs) [6, 18, 19], especially in sub-Saharan Africa (SSA) [20, 21]. The current literature showcases barriers to access and utilization of routine and emergency maternal health services in SSA [22–24]. This includes physical, topographical and financial barriers, as well as lack of knowledge, trust and awareness of available services. Consequently, the SSA region accounts for the highest burden of maternal deaths, carrying 68% of maternal deaths worldwide every year (UNICEF, 2019) [25, 26]. Nevertheless, most SSA countries have seen some progress in maternal health outcomes in the last 15 years, compared to previous periods [27–30]. Most notably, the region had a 45% reduction in the maternal mortality ratio between 1990 and 2015 [27], and varied improvements in access to evidence-based antenatal, childbirth and postnatal care services [28–30]. This progress is partly attributable to transformative, technological innovations, that have helped to mitigate certain gaps in healthcare systems [31]. Digital health technology has made major inroads throughout SSA, and it is increasingly becoming an integral component of healthcare in many communities [31, 32] Mobile health (mHealth) technology is a type of digital health that delivers care through mobile technology including mobile phones, tablets or smart watches and has shown immense potential to bridge existing gaps in maternal and essential healthcare service delivery in developing regions [32–34].

Many governments and leading national organizations have been enthusiastic about the utility of mHealth devices and applications, as scalable tools, to provide effective, efficient, safe and personalized care to service users [32, 33, 35]. In addition, mHealth devices and applications can be implemented at all levels of a health system, including the home, the community, primary, secondary and tertiary level care [36]. When applied to maternal health, mHealth devices and applications have helped to mitigate geographic, infrastructural and human resource challenges, as well as to improve health provider and patient education [37–39]. In the last decade, mobile phone adoption has risen rapidly in SSA [40, 41], a region that has emerged as a major arena for innovative mHealth interventions [42–44]. Such interventions have demonstrated the utility of mobile phones for reaching hard-to-reach locations with limited infrastructure [38, 42], and for strengthening communication throughout different levels of a health system [45]. This is significant as global health initiatives targeting maternal health often fail to reach women in underserved rural and remote areas [36].

Over the past 15 years, a large number of mobile phone interventions have been developed and placed in the market [42–44]. In accordance, there has been an increase in publications that describe the development, implementation, feasibility and impact of mobile phone interventions on health services. Several reviews have synthesized the emerging evidence on mHealth interventions, including mobile phone interventions, targeting maternal healthcare in SSA and other LMIC regions [34, 37, 39, 42, 46–61]. The majority of these reviews are systematic reviews that assess and collate evidence on the feasibility, appropriateness, or effectiveness of mobile and other mHealth interventions [37, 39, 42, 46, 51, 56–59]. Four recent scoping reviews examined the scope, coverage and/or conceptualization of mHealth interventions in LMICs [50, 52, 60, 61]. However, these reviews focus on a specific population, such as community health workers [61], a specific outcome, such as behavior change [52], or a specific context, such as the provision of services during disruptive events [50]. There is currently no extensive mapping of the available literature on mobile phone interventions that aim to improve accessibility and utilization of maternal care services across the continuum of maternity care in SSA.

As such, a scoping review is needed to examine the rapidly emerging evidence on mobile phone interventions developed to improve the delivery of maternal care services. The purpose of this scoping review is therefore to examine the nature and extent of mobile phone interventions used to improve maternal healthcare services in SSA. The review will determine the volume and scope of the interventions, and provide an overview of their targeted users, features and functionalities, and degree of integration within maternal healthcare provision systems. The review will also highlight the gaps and challenges in the development and implementation of these interventions, as well as the best practices for addressing these problems. Therefore, the findings of this scoping review will inform future research, and the developments and implementation of new mobile interventions, and/or the refinements to existing mobile phone interventions.

### Review question

What mobile phone interventions are available to assist the provision of maternal health services in sub-Saharan Africa?

## Methods

This review protocol is reported in accordance with the reporting guidance provided by the Preferred Reporting Items for Systematic Reviews and Meta-Analyses Protocols (PRISMA-P) criteria [62]. This protocol is registered with the Open Science Framework [63]. The scoping review will be guided by the JBI approach to the conduct of scoping reviews described in the JBI Evidence Synthesis Manual [64]. We will use the PRISMA-ScR to guide the development, conduct and reporting of this review [65].

### Patient and public involvement

Public consultation took place during two (2) global health conferences, one in Geneva, Switzerland and another in Lisbon, Portugal, in 2022. The objective of these consultations was to engage a broad audience and to get additional feedback to inform our review questions and nature of data to be extracted. During these consultations, global health practitioners and researchers expressed concern and surprise by the lack of scoping review of digital and mobile health interventions addressing maternal mortality in sub–Saharan Africa. A definite gap in the research was established and potential use for research findings for evidence-based policy implementation was acknowledged. Further, there was concern expressed over the current number of digital health interventions in existence and the lack of coordination, collaboration, and interoperability amongst implementers of these interventions. As such, this scoping review may further assist policy makers to establish a coordinated approach to the implementation of mHealth interventions addressing maternal mortality in SSA in order to align practitioners and increase efficiencies of political, technical and financial investments.

### Inclusion criteria

#### Participants

Studies on the provision of maternal health services for women during pregnancy, childbirth and puerperium through the use of mobile phones. This will include studies involving health service providers such as doctors (including but not limited to obstetricians and gynecologists) nurses, midwives and community health workers (CHWs).

#### Concept

The concept for this review is the use of mobile phones to assist delivery of maternal health services. Mobile phones include basic phones (with no computing or internet capacity), smart phones and other mobile telephone device with applications and functionalities that can be used to provide services that link the service provider (doctor, nurses/midwives, CHW etc) to clients without a face-to-face encounter. By maternal health services, we particularly refer to services that are provided to a woman during the periods of pregnancy, childbirth and puerperium [66]. Studies that report the use of mobile phones to deliver any service related to the above-mentioned core services will be considered.

#### Context

This review will consider studies that have been conducted in both health care facility and community settings in sub-Saharan Africa. Studies conducted on African populations out of the African continent will not be considered given that their inclusion may mask critical contextual constraints that needs to be considered during data analysis. The rationale for sub–Saharan Africa is based on the current burden of maternal mortality [25, 26].

#### Types of evidence sources

Studies that used quantitative, qualitative and mixed methods designs will be considered for inclusion. Quantitative study designs of interest will include experimental, quasi-experimental (RCTs and non-RCTs), interrupted time series, and pre-post-test studies. Observational studies such as prospective and retrospective cohort studies, cross sectional and case control studies will be identified. Observational studies that are descriptive in nature such as individual case reports and case series, cross sectional studies will also be considered for inclusion.

Qualitative study designs for consideration will not be limited to phenomenology, action research, grounded theory, qualitative description, ethnography and feminist research. Beyond primary research studies, we will consider evidence emanating from systematic reviews, case reports, practice guidelines, text and comment/opinion papers, grey literature, websites and blogs for eligibility.

### Information sources and search

Search strategies will be developed by an information specialist (KF) and peer reviewed using the PRESS guideline [67]. The search will be conducted in: MEDLINE(R) ALL (OvidSP), Embase (OvidSP), CINAHL (EBSCOHost), APA PsycInfo (OvidSP), Cochrane Central Register of Controlled Trials (OvidSP), Global Health (EBSCOHost), African Index Medicus, Web of Science, and Applied Social Sciences Index & Abstracts (ProQuest). Each database will be searched from their inception for the concept of “maternal health”, “mobile devices” and “Sub-Saharan Africa” using a combination of subject headings and keywords. Drafting the search strategy was informed by a Cochrane review for the concept of mobile devices, [55] and by consulting the search method from the Cochrane Pregnancy and Childbirth’s Trials Register [68] for the concept of maternal health. No search filters or language limits will be used. In addition, no publication date limits will be applied. The Medline search strategy can be found in the extended data. Additionally, the reference list of all identified sources will be searched for additional studies. Authors of primary studies will be contacted for additional information if necessary. A strategy will be developed to search for unpublished studies and grey literature from databases such as: ProQuest Dissertation and Theses and Google Scholar, websites and digital repositories (mHealth/digital health).

### Source of evidence selection

All database results will be sent to Covidence (Veritas Health Innovation Ltd.), where duplicate records will be removed automatically. Following a pilot test, titles and abstracts will then be screened by two or more independent reviewers for assessment against the inclusion criteria for the review. Potentially relevant studies will be retrieved in full, and their citation details will be imported into the JBI System for the Unified Management, Assessment and Review of Information (JBI SUMARI) [64].

Two or more independent reviewers will assess the full text in detail against the inclusion criteria. Any evidence source that does not meet the inclusion criteria will be excluded and reasons for exclusion will be reported in the final review report. Any disagreement that arises between the reviewers at each stage of the selection process will be resolved through discussion or with an additional reviewer. The results of the search and the study inclusion process will be reported in full in the final scoping review and presented in a Preferred Reporting Items for Systematic Reviews and Meta-analyses extension for scoping review (PRISMA-ScR) flow diagram [65].

### Data extraction

Two independent reviewers will extract data from all included studies using a data extraction tool developed by the review authors (see in extended data). Any disagreement will be resolved by a third reviewer. The tool will be tested during a pilot trial and any relevant modifications will be made and detailed in the review. Any disagreement that arises between the reviewers at each stage of the selection process will be resolved through discussion or with an additional reviewer. If appropriate, authors of papers will be contacted to request missing or additional data, where required.

Data to be extracted will include details such as title, authors, objective, methodology, population characteristics, concept, context, type of evidence source, interventions (including name, type, description, timing of evaluation if available), study results, and gaps and recommendations. In the event of any missing or ambiguous data from a study, the corresponding author of the study will be contacted to retrieve missing or additional data.

### Data analysis and presentation

Data will be presented using either tables, charts, maps or a combination of these formats depending on the type of results we retrieve. A narrative summary will accompany the tabulated and/or charted results and will describe how the results relate to the review objective and question/s. The first content of the presentation will include details on author and year of publication, type of source, setting, study design, geographic location, delivery format and population. The second set of presentation will dwell on the various existing mobile phones and associated applications used to deliver maternal health services in Africa, services provided, functionalities, effectiveness, impact, challenges, opportunities, gaps, and/or recommendations.

## Discussion

While most SSA countries have made substantive progress in maternal health outcomes in the last 15 years [27–30], progress has been slow. However, transformative, technological innovations, have been reported to have helped to mitigate certain gaps in healthcare systems [31]. Particularly, digital health technology has made major contributions throughout SSA, and it is increasingly becoming an integral component of healthcare in many communities [31, 32]. While mHealth technology has shown immense potential to bridge existing gaps in maternal and essential healthcare service delivery in developing regions [33, 34], there still remains a gap in evidence on the nature and extent of mobile phone interventions available to assist the provision of maternal health services in sub-Saharan Africa.

An understanding of the nature and extent of mobile phone interventions available to assist the provision of maternal health services is critical to examining the rapidly emerging evidence on mobile phone interventions developed to improve the delivery of maternal care services. Through this scoping review, it is hoped that the volume and scope of the mobile phone interventions, including their targeted users and functionalities will be well understood. Additionally, we hope that through this review, some gaps and challenges in the development and implementation of these interventions, as well as the best practices for addressing these problems will be highlighted and potentially inform future research, and the developments and implementation of new mobile interventions.

## Supporting information

S1 Checklist PRISMA-P (Preferred Reporting Items for Systematic review and Meta-Analysis Protocols) 2015 checklist: Recommended items to address in a systematic review protocol*. (DOC)

S1 File Appendix A: Search strategy. (DOC)

## References

[1] World Health Organization. Maternal health. Accessed April 9, 2022. https://www.who.int/health-topics/maternal-health#tab=tab_1

[2] BlackRE, WalkerN, LaxminarayanR, TemmermanM. Reproductive, Maternal, Newborn, and Child Health: Key Messages of This Volume. In: BlackRE, LaxminarayanR, TemmermanM, et al. editors., eds. *Reproductive*, *Maternal*, *Newborn*, *and Child Health*: *Disease Control Priorities*, *Third Edition (Volume 2)*. Vol 2. 3rd ed. The International Bank for Reconstruction and Development / The World Bank; 2016. doi: 10.1596/978-1-4648-0348-2_CH127227235

[3] Mehedi Hasan M, Soares Magalhaes RJ, Ahmed S, et al. Meeting the global target in reproductive, maternal, newborn, and child health care services in low- And middle-income countries. *Glob Heal Sci Pract*. 2021;8(4):654–665. doi: 10.9745/GHSP-D-20-00097/-/DCSUPPLEMENTAL PMC7784071 33361233

[4] Mahler H. The safe motherhood initiative: a call to action. *Lancet*. 1987;1(8534):668–670. doi: 10.1016/s0140-6736(87)90423-5 2882090

[5] United Nations. Millennium Development Goals (MDGs): Progress Report on Health-related MDGs. Published February 19, 2018. Accessed February 5, 2022. https://www.who.int/news-room/fact-sheets/detail/millennium-development-goals-(mdgs)

[6] Boerma T, Requejo J, Victora CG, et al. Countdown to 2030: tracking progress towards universal coverage for reproductive, maternal, newborn, and child health. *Lancet*. 2018;391(10129):1538–1548. doi: 10.1016/S0140-6736(18)30104-1 29395268

[7] United Nations. Transforming our world: the 2030 Agenda for Sustainable Development | Department of Economic and Social Affairs. United Nations. Department of Economic and Social Affairs. Published October 21, 2015. Accessed February 5, 2022. https://sdgs.un.org/2030agenda

[8] Every Woman Every Child. Global Strategy for Women’s and Children’s health. Published 2010. Accessed February 5, 2022. https://www.who.int/pmnch/knowledge/publications/fulldocument_globalstrategy/en/

[9] Every Woman Every Child. The Global Strategy For Women’s, Children’s And Adolescents’ Health (2016–2030). Every Woman Every Child. Published 2015. Accessed February 5, 2022. https://www.who.int/life-course/partners/global-strategy/globalstrategyreport2016-2030-lowres.pdf

[10] World Health Organization. *Ending Preventable Maternal Mortality (EPMM)*: *A Renewed Focus for Improving Maternal and Newborn Health and Well-Being*.; 2021. Accessed April 9, 2022. https://cdn.who.int/media/docs/default-source/mca-documents/maternal-nb/ending-preventable-maternal-mortality_epmm_brief-230921.pdf?sfvrsn=f5dcf35e_5

[11] Arregoces L, Daly F, Pitt C, et al. Countdown to 2015: Changes in official development assistance to reproductive, maternal, newborn, and child health, and assessment of progress between 2003 and 2012. *Lancet Glob Heal*. 2015;3(7):e410–e421. doi: 10.1016/S2214-109X(15)00057-1 26087987

[12] Brizuela V, Tunçalp Ö. Global initiatives in maternal and newborn health. *Obstet Med*. 2017;10(1):25. doi: 10.1177/1753495X16684987 28491127 PMC5405944

[13] Elavarasan RM, Pugazhendhi R, Shafiullah GM, et al. Impacts of COVID-19 on Sustainable Development Goals and effective approaches to maneuver them in the post-pandemic environment. *Environ Sci Pollut Res*. 2022;29(23):33957–33987. doi: 10.1007/s11356-021-17793-9 35032263 PMC8760582

[14] Nyamtema AS, Urassa DP, van Roosmalen J. Maternal health interventions in resource limited countries: A systematic review of packages, impacts and factors for change. *BMC Pregnancy Childbirth*. 2011;11(1):1–12. doi: 10.1186/1471-2393-11-30 21496315 PMC3090370

[15] Jokhio AH, Winter HR, Cheng KK. An intervention involving traditional birth attendants and perinatal and maternal mortality in Pakistan. *N Engl J Med*. 2005;352(20):2091–2099. doi: 10.1056/NEJMsa042830 15901862

[16] Lori JR, Munro ML, Rominski S, et al. Maternity waiting homes and traditional midwives in rural Liberia. *Int J Gynaecol Obstet*. 2013;123(2):114–118. doi: 10.1016/j.ijgo.2013.05.024 23992657 PMC3795996

[17] Balakrishnan R, Gopichandran V, Chaturvedi S, Chatterjee R, Mahapatra T, Chaudhuri I. Continuum of Care Services for Maternal and Child Health using mobile technology—a health system strengthening strategy in low and middle income countries. *BMC Med Inform Decis Mak*. 2016;16(1):1–8. doi: 10.1186/s12911-016-0326-z 27387548 PMC4937606

[18] World Health Organization. *State of Inequality- Reproductive*, *Maternal*, *Newborn and Child Health*.; 2015. Accessed April 9, 2022. https://www.who.int/docs/default-source/gho-documents/health-equity/state-of-inequality/state-of-inequality-reproductive-maternal-new-born-and-child-health.pdf?sfvrsn=f4034289_2

[19] Amouzou A, Jiwani SS, Da Silva ICM, Carvajal-Aguirre L, Maïga A, Vaz LME. Closing the inequality gaps in reproductive, maternal, newborn and child health coverage: slow and fast progressors. *BMJ Glob Heal*. 2020;5(1):e002230. doi: 10.1136/bmjgh-2019-002230 32133181 PMC7042586

[20] Faye CM, Wehrmeister FC, Melesse DY, et al. Large and persistent subnational inequalities in reproductive, maternal, newborn and child health intervention coverage in sub-Saharan Africa. *BMJ Glob Heal*. 2020;5(1):e002232. doi: 10.1136/bmjgh-2019-002232 32133183 PMC7042572

[21] Yaya S, Ghose B. Global Inequality in Maternal Health Care Service Utilization: Implications for Sustainable Development Goals. *Heal Equity*. 2019;3(1):154. doi: 10.1089/heq.2018.0082 31289773 PMC6608688

[22] Geleto A, Chojenta C, Musa A, Loxton D. Barriers to access and utilization of emergency obstetric care at health facilities in sub-Saharan Africa: A systematic review of literature 11 Medical and Health Sciences 1117 Public Health and Health Services. *Syst Rev*. 2018;7(1):1–14. doi: 10.1186/S13643-018-0842-2/FIGURES/3 30424808 PMC6234634

[23] Dahab R, Sakellariou D. Barriers to Accessing Maternal Care in Low Income Countries in Africa: A Systematic Review. *Int J Environ Res Public Health*. 2020;17(12):4292. doi: 10.3390/ijerph17124292 32560132 PMC7344902

[24] Fantaye AW, Gunawardena N, Yaya S. Preferences for formal and traditional sources of childbirth and postnatal care among women in rural Africa: A systematic review. *PLoS One*. 2019;14(9):e0222110. doi: 10.1371/journal.pone.0222110 31553722 PMC6760778

[25] UNICEF. Maternal mortality. Published September 2019. Accessed February 5, 2022. https://data.unicef.org/topic/maternal-health/maternal-mortality/#:~:text=Sub-Saharan Africans suffer from,200%2C000 maternal deaths a year.&text = The global lifetime risk of,100%2C to 1 in 190.

[26] World Health Organization. *Trends in Maternal Mortality 2000 to 2017*: *Estimates by WHO*, *UNICEF*, *UNFPA*, *World Bank Group and the United Nations Population Division*: *Executive Summary*.; 2019. Accessed April 23, 2022. https://apps.who.int/iris/handle/10665/327595

[27] WHO, UNICEF, UNFPA, World Bank Group, United Nations Population Division. *Trends in Maternal Mortality*: *1990 to 2015 | United Nations Population Fund*.; 2015. Accessed February 5, 2022. http://apps.who.int/iris/bitstream/10665/193994/1/WHO_RHR_15.23_eng.pdf

[28] Zuñiga JA, García A, Kyololo OM, et al. Increasing utilisation of skilled attendants at birth in sub-Saharan Africa: A systematic review of interventions. *Int J Nurs Stud*. 2021;120. doi: 10.1016/j.ijnurstu.2021.103977 34144356

[29] Alam N, Hajizadeh M, Dumont A, Fournier P. Inequalities in maternal health care utilization in sub-Saharan African countries: a multiyear and multi-country analysis. *PLoS One*. 2015;10(4):e0120922. doi: 10.1371/journal.pone.0120922 25853423 PMC4390337

[30] Doctor H V., Nkhana-Salimu S, Abdulsalam-Anibilowo M. Health facility delivery in sub-Saharan Africa: Successes, challenges, and implications for the 2030 development agenda. *BMC Public Health*. 2018;18(1):765. doi: 10.1186/s12889-018-5695-z 29921275 PMC6011205

[31] Engmann CM, Khan S, Moyer CA, Coffey PS, Bhutta ZA. Transformative Innovations in Reproductive, Maternal, Newborn, and Child Health over the Next 20 Years. *PLOS Med*. 2016;13(3):e1001969. doi: 10.1371/journal.pmed.1001969 26933951 PMC4774905

[32] Holst C, Sukums F, Radovanovic D, Ngowi B, Noll J, Winkler AS. Sub-Saharan Africa—the new breeding ground for global digital health. *Lancet Digit Heal*. 2020;2(4):e160–e162. doi: 10.1016/S2589-7500(20)30027-3 33328076

[33] World Health Organization. Digital health. Published May 2018. Accessed February 5, 2022. https://apps.who.int/gb/ebwha/pdf_files/WHA71/A71_R7-en.pdf

[34] Manyazewal T, Woldeamanuel Y, Blumberg HM, Fekadu A, Marconi VC. The potential use of digital health technologies in the African context: a systematic review of evidence from Ethiopia. *npj Digit Med 2021 41*. 2021;4(1):1–13. doi: 10.1038/s41746-021-00487-4 34404895 PMC8371011

[35] Murray E, Hekler EB, Andersson G, et al. Evaluating digital health interventions: key questions and approaches. *Am J Prev Med*. 2016;51(5):843. doi: 10.1016/j.amepre.2016.06.008 27745684 PMC5324832

[36] World Health Organization. *A Policy Guide for Implementing Essential Interventions for Reproductive*, *Maternal*, *Newborn and Child Health (‎RMNCH)‎*: *A Multisectoral Policy Compendium for RMNCH*.; 2014. Accessed February 6, 2022. https://www.who.int/pmnch/knowledge/publications/policy_compendium.pdf

[37] Chen H, Chai Y, Dong L, Niu W, Zhang P. Effectiveness and Appropriateness of mHealth Interventions for Maternal and Child Health: Systematic Review. *JMIR mHealth uHealth*. 2018;6(1). doi: 10.2196/mhealth.8998 29317380 PMC5780618

[38] WHO Global Observatory for eHealth. *MHealth*: *New Horizons for Health through Mobile Technologies*: *Second Global Survey on EHealth*.; 2011. Accessed February 6, 2022. https://apps.who.int/iris/handle/10665/44607

[39] Maliwichi P, Chigona W, Sowon K. Appropriation of mHealth Interventions for Maternal Health Care in Sub-Saharan Africa: Hermeneutic Review. *JMIR mHealth uHealth*. 2021;9(10):e22653. doi: 10.2196/22653 34612835 PMC8529477

[40] Global System for Mobile Communications Association. *The Mobile Economy- Sub-Saharan Africa 2015*.; 2016. Accessed April 9, 2022. https://www.gsma.com/subsaharanafrica/resources/the-mobile-economy-sub-saharan-africa-2015

[41] Global System for Mobile Communications Association. *The Mobile Economy Sub-Saharan Africa 2020*.; 2021. Accessed April 9, 2022. https://www.gsma.com/mobileeconomy/wp-content/uploads/2020/09/GSMA_MobileEconomy2020_SSA_Eng.pdf

[42] Lee SH, Nurmatov UB, Nwaru BI, Mukherjee M, Grant L, Pagliari C. Effectiveness of mHealth interventions for maternal, newborn and child health in low–and middle–income countries: Systematic review and meta–analysis. *J Glob Health*. 2016;6(1). doi: 10.7189/jogh.06.010401 26649177 PMC4643860

[43] Global System for Mobile Communications. *GiftedMom—Using Mobile Channels to Democratise and Reinvent Access to Maternal Healthcare in Francophone Africa*.; 2019. Accessed April 9, 2022. https://www.gsma.com/mobilefordevelopment/wp-content/uploads/2019/08/GiftedMom-Using-mobile-channels-to-democratise-and-reinvent-access-to-maternal-healthcare-in-Francophone-Africa_SINGLE.pdf

[44] Zero Mothers Die. ZMD App—Zero Mothers Die. Zero Mothers Die Partnership Consortium. Published 2017. Accessed April 9, 2022. http://www.zeromothersdie.org/zmd-app.html

[45] Noordam AC, Kuepper BM, Stekelenburg J, Milen A. Improvement of maternal health services through the use of mobile phones. *Trop Med Int Heal*. 2011;16(5):622–626. doi: 10.1111/j.1365-3156.2011.02747.x 21342374

[46] Dol J, Richardson B, Tomblin Murphy G, Aston M, McMillan D, Campbell-Yeo M. Impact of mobile health (mHealth) interventions during the perinatal period for mothers in low- and middle-income countries: A systematic review. *JBI Database Syst Rev Implement Reports*. 2019;17(8):1634–1667. doi: 10.11124/JBISRIR-2017-004022 31404051

[47] Bervell B, Al-Samarraie H. A comparative review of mobile health and electronic health utilization in sub-Saharan African countries. *Soc Sci Med*. 2019;232:1–16. doi: 10.1016/j.socscimed.2019.04.024 31035241

[48] Manyati TK, Mutsau M. A systematic review of the factors that hinder the scale up of mobile health technologies in antenatal care programmes in sub-Saharan Africa. https://doi.org/101080/2042133820201765479. 2020;13(1):125–131.

[49] Kabongo EM, Mukumbang FC, Delobelle P, Nicol E. Explaining the impact of mHealth on maternal and child health care in low- and middle-income countries: a realist synthesis. *BMC Pregnancy Childbirth*. 2021;21(1):1–13. doi: 10.1186/S12884-021-03684-X/FIGURES/5 33750340 PMC7941738

[50] World Health Organization. *Scoping Review of Interventions to Maintain Essential Services for Maternal*, *Newborn*, *Child and Adolescent Health and Older People during Disruptive Events*.; 2021. Accessed February 6, 2022. https://apps.who.int/iris/rest/bitstreams/1386307/retrieve

[51] Feroz A, Perveen S, Aftab W. Role of mHealth applications for improving antenatal and postnatal care in low and middle income countries: a systematic review. *BMC Health Serv Res*. 2017;17(1). doi: 10.1186/s12913-017-2664-7 29115992 PMC5678803

[52] Mildon A, Sellen D. Use of mobile phones for behavior change communication to improve maternal, newborn and child health: a scoping review. *J Glob Health*. 2019;9(2):020425. doi: 10.7189/jogh.09.020425 31893032 PMC6925966

[53] Avoka CK, McArthur E, Banke-Thomas A. Interventions to improve obstetric emergency referral decision making, communication and feedback between health facilities in sub-Saharan Africa: A systematic review. *Trop Med Int Heal*. 2022;27(5):494–509. doi: 10.1111/tmi.13747 35322914 PMC9321161

[54] Saronga NJ, Burrows T, Collins CE, Ashman AM, Rollo ME. mHealth interventions targeting pregnancy intakes in low and lower-middle income countries: Systematic review. *Matern Child Nutr*. 2019;15(2):e12777. doi: 10.1111/mcn.12777 30609297 PMC7199073

[55] Agarwal S, Glenton C, Tamrat T, et al. Decision-support tools via mobile devices to improve quality of care in primary healthcare settings. *Cochrane database Syst Rev*. 2021;7(7):CD012944. doi: 10.1002/14651858.CD012944.pub2 34314020 PMC8406991

[56] Sondaal SFV, Browne JL, Amoakoh-Coleman M, et al. Assessing the Effect of mHealth Interventions in Improving Maternal and Neonatal Care in Low- and Middle-Income Countries: A Systematic Review. *PLoS One*. 2016;11(5):e0154664. doi: 10.1371/journal.pone.0154664 27144393 PMC4856298

[57] Amoakoh-Coleman M, Borgstein ABJ, Sondaal SFV, et al. Effectiveness of mHealth Interventions Targeting Health Care Workers to Improve Pregnancy Outcomes in Low- and Middle-Income Countries: A Systematic Review. *J Med Internet Res*. 2016;18(8):e226. doi: 10.2196/jmir.5533 27543152 PMC5010646

[58] Colaci D, Chaudhri S, Vasan A. mHealth Interventions in Low-Income Countries to Address Maternal Health: A Systematic Review. *Ann Glob Heal*. 2016;82(5):922–935. doi: 10.1016/j.aogh.2016.09.001 28283147

[59] Ahmed MAA, Gagnon M-P, Hamelin-Brabant L, Mbemba GIC, Alami H. A mixed methods systematic review of success factors of mhealth and telehealth for maternal health in Sub-Saharan Africa. *mHealth*. 2017;3:22–22. doi: 10.21037/mhealth.2017.05.04 28736731 PMC5505928

[60] Adepoju IOO, Albersen BJA, De Brouwere V, van Roosmalen J, Zweekhorst M. mHealth for Clinical Decision-Making in Sub-Saharan Africa: A Scoping Review. *JMIR mHealth uHealth*. 2017;5(3):e38. doi: 10.2196/mhealth.7185 28336504 PMC5383806

[61] Winters N, Langer L, Geniets A. Scoping review assessing the evidence used to support the adoption of mobile health (mHealth) technologies for the education and training of community health workers (CHWs) in low-income and middle-income countries. *BMJ Open*. 2018;8(7):e019827. doi: 10.1136/bmjopen-2017-019827 30061430 PMC6067337

[62] Moher D, Shamseer L, Clarke M, et al. Preferred reporting items for systematic review and meta-analysis protocols (PRISMA-P) 2015 statement. *Syst Rev*. 2015;4(1):1. doi: 10.1186/2046-4053-4-1 25554246 PMC4320440

[63] Fantaye AW, Constantine A, Jacobs C, et al. *Utilisation of Mobile Phone Interventions to Improve the Delivery of Maternal Health Services in Sub-Saharan Africa*: *A Scoping Review Protocol*. OSF; 2022. doi: 10.17605/OSF.IO/SZKY3 PMC10917244 38446819

[64] Peters M, Godfrey C, McInerney P, Munn Z, Trico A, Khalil H. Chapter 11: Scoping Reviews. JBI Manual for Evidence Synthesis. doi: 10.46658/JBIMES-20-12

[65] Tricco AC, Lillie E, Zarin W, et al. PRISMA extension for scoping reviews (PRISMA-ScR): Checklist and explanation. *Ann Intern Med*. 2018;169(7):467–473. doi: 10.7326/M18-0850 30178033

[66] World Health Organization. *WHO Recommendations on Maternal Health*: *Guidelines Approved by the WHO Guidelines Review Committee*.; 2017. Accessed June 25, 2022. http://apps.who.int/iris/bitstream/handle/10665/259268/WHO-MCA-17.10-eng.pdf?sequence=1. 40455892

[67] McGowan J, Sampson M, Salzwedel DM, Cogo E, Foerster V, Lefebvre C. PRESS Peer Review of Electronic Search Strategies: 2015 Guideline Statement. *J Clin Epidemiol*. 2016;75:40–46. doi: 10.1016/J.JCLINEPI.2016.01.021 27005575

[68] Cochrane Pregnancy and Childbirth. Cochrane Pregnancy and Childbirth’s Trials Register. Accessed June 25, 2022. https://pregnancy.cochrane.org/pregnancy-and-childbirth-groups-trials-register

