# Supplementary material for: Utilisation of mobile phone interventions to improve the delivery of maternal health services in sub-Saharan Africa: A scoping review protocol
Source: PLoS One. 2024 Mar 6;19(3):e0295437. doi: 10.1371/journal.pone.0295437 (PMC10917244; doi:10.1371/journal.pone.0295437)
Supplement: S1 File — (DOC) [file pone.0295437.s002.doc]

**Supplementary Files**

**Appendix A: Search strategy**

| Ovid MEDLINE(R) ALL <1946 to June 13, 2022> | | |
| --- | --- | --- |
|  |  |  |
| 1 | exp Maternal Health Services/ or Maternal Health/ or Maternal Welfare/ | 61862 |
| 2 | Prenatal Education/ | 327 |
| 3 | exp Pregnancy/ | 970731 |
| 4 | exp Pregnancy Complications/ | 460632 |
| 5 | peripartum period/ or exp postpartum period/ or exp pregnancy trimesters/ | 116277 |
| 6 | Pregnant Women/ | 12337 |
| 7 | Obstetrics/ or exp Delivery, Obstetric/ | 109192 |
| 8 | exp Breast Feeding/ or Bottle Feeding/ | 43558 |
| 9 | (matern* or pregnan* or gestation*).ti,ab,kf. | 844932 |
| 10 | (expect* adj3 (mother* or mom? or child* or baby or babies)).ti,ab,kf. | 5817 |
| 11 | (prenatal* or perinatal* or antenatal* or postnatal* or postpartum* or post-partum* or pre-partum* or intrapartum*).ti,ab,kf. | 372383 |
| 12 | (Peripartum* or puerperium* or puerperal*).ti,ab,kf. | 20588 |
| 13 | Obstetric*.ti,ab,kf. | 112094 |
| 14 | Parturition*.ti,ab,kf. | 15872 |
| 15 | (Lactat* or breastfeed* or breast feed* or breast milk or milk expression* or ((bottle* or formula*) adj3 feed*)).ti,ab,kf. | 229843 |
| 16 | (Childbirth* or child-bearing).ti,ab,kf. | 24609 |
| 17 | ((preterm or premature or induced) adj3 (labour* or labor*)).ti,ab,kf. | 16146 |
| 18 | ((labour* or labor* or deliver*) adj3 (complication* or pain*)).ti,ab,kf. | 12875 |
| 19 | birth*.ti,ab,kf. | 378429 |
| 20 | (caesarean or cesarean or caesarian or cesarian or cesarien or caesarien).ti,ab,kf. | 70158 |
| 21 | (eclamp* or preeclamp* or (pre adj1 eclamp*)).ti,ab,kf. | 41236 |
| 22 | (amniocentes* or episiotom*).ti,ab,kf. | 11602 |
| 23 | placenta*.ti,ab,kf. | 113134 |
| 24 | (prepregnancy or pre-pregnancy or post-pregnancy or postpregnancy or preconception* or pre-conception* or postconception* or post-conception* or periconception* or peri-conception*).ti,ab,kf. | 25456 |
| 25 | or/1-24 | 1788624 |
| 26 | exp Cell Phone/ or mobile applications/ or Remote Consultation/ | 32265 |
| 27 | ((cell* or mobile*) adj1 (phone* or telephone* or technolog* or device*)).ti,ab,kf. | 26128 |
| 28 | (handheld or "hand‐held").ti,ab,kf. | 7835 |
| 29 | (smartphone* or "smart-phone*" or cellphone* or mobiles).ti,ab,kf. | 20841 |
| 30 | (samsung or nokia).ti,ab,kf. | 1470 |
| 31 | (windows adj3 (mobile* or phone*)).ti,ab,kf. | 57 |
| 32 | android.ti,ab,kf. | 3347 |
| 33 | (iphone* or "i‐phone*").ti,ab,kf. | 1114 |
| 34 | (mhealth or "m‐health" or "mobile health" or ehealth or "e‐health" or "electronic health" or "digital health" or uhealth or u-health).ti,ab,kf. | 44712 |
| 35 | (((text* or short or voice or multimedia or "multi‐media" or electronic or instant) adj1 messag*) or instant messenger).ti,ab,kf. | 7393 |
| 36 | (texting or texted or texter* or ((sms or mms) adj (service* or messag*)) or interactive voice response* or IVR or voice call* or callback* or voice over internet or VOIP).ti,ab,kf. | 4244 |
| 37 | ((online* or "on‐line*" or virtual* or internet* or web* or distanc* or remote* or mobile* or electronic* or computer*) adj3 consult*).ti,ab,kf. | 3826 |
| 38 | mobile app*.ti,ab,kf. | 8156 |
| 39 | or/26-38 | 114678 |
| 40 | exp "Africa South of the Sahara"/ | 241357 |
| 41 | ("Sub Saharan Africa*" or "Central Africa*" or "Eastern Africa*" or "Southern Africa*" or "Western Africa*" or Angola* or Benin* or Botswana* or Burkina Faso* or Burundi* or Cameroon* or Cape Verde* or Central African Republic* or Chad* or Comoros* or Congo* or "Cote d'ivoire" or Djibouti* or Equatorial Guinea* or Eritrea* or Ethiopia* or Gabon* or Gambia* or Ghana* or Guinea* or "Guinea bissau*" or Ivory Coast* or Kenya* or Lesotho* or Liberia* or Madagascar* or Malawi* or Mali* or Mauritania* or Mauritius* or Mozambique* or Namibia* or Niger* or Nigeria* or Reunion* or Rwanda* or Senegal* or Seychelles* or Sierra Leone* or Somalia* or South Africa* or Sudan* or Swaziland* or Tanzania* or Togo* or Uganda* or Zambia* or Zimbabwe*).ti,ab,kf. | 1091103 |
| 42 | or/40-41 | 1137725 |
| 43 | 25 and 39 and 42 | 626 |
